# Optimal Operation for Electricity–Hydrogen Integrated Energy System Accounting for Dynamic Traits of Proton Exchange Membrane Electrolyzer

**DOI:** 10.3390/membranes16040127

**Published:** 2026-03-31

**Authors:** Chengbo Mao, Chaoping Rao, Jitao Liang, Jiahao Wang, Peirong Ji, Yi Zheng

**Affiliations:** 1 XJ Electric Corporation, Xuchang 461000, China; maochengbo_xj@163.com (C.M.); 15290987298@163.com (J.L.); 2 Department of Mechanical and Electrical Engineering, Wuhan Qingchuan University, Wuhan 430204, China; 3 School of Electrical and Automation, Wuhan University, Wuhan 430072, China; 4 College of Electrical and New Energy, China Three Gorges University, Yichang 443002, China; jipeirongwf@126.com; 5 School of Mechanical and Automotive Engineering, Qingdao University of Technology, Qingdao 266520, China

**Keywords:** dynamic traits, IES, proton exchange membrane, optimal operation, loss of life cost

## Abstract

The proton exchange membrane (PEM) electrolyzer is vital for converting surplus renewable energy (RE) into hydrogen, underpinning the efficient and stable operation of the electric–hydrogen system. However, frequent start–stop cycles and load variations accelerate the degradation of proton exchange membranes and catalyst layers, incurring significant lifetime costs that existing studies ignore. To explore how the PEM electrolyzer’s dynamic traits impact system performance, we introduce an optimized operation approach for the electricity–hydrogen integrated energy system (IES) that incorporates these dynamic features and the novel Loss of Life Cost (LLC) model. Initially, to rectify the inadequacy in modeling the PEM electrolyzer within the current electricity–hydrogen IES operational framework, we integrate its dynamic characteristics based on electrochemical properties and establish a quantitative relationship between operational cycles and degradation costs. This enhanced model accurately reflects how operational conditions affect the electrolyzer’s hydrogen production efficiency and lifetime consumption, enabling precise performance simulation and economic assessment. This, in turn, promotes high-quality renewable energy utilization via hydrogen production while ensuring asset longevity, meeting the rising demand for hydrogen energy applications. Building on this, we further factor in constraints related to diverse energy conversion and safe operation within the electricity–hydrogen IES, as well as the operational limits of hydrogen fuel cells, various energy storage (ES) options, cogeneration units, and other pertinent equipment, aiming to minimize the system’s total daily costs (operational plus degradation costs). Consequently, we develop an optimization operation model for the electricity–hydrogen IES that accounts for the PEM electrolyzer’s dynamic characteristics and degradation economics. Finally, through simulation examples validated against published experimental data, we comprehensively analyze how the PEM electrolyzer’s dynamic traits influence system operation, confirming the effectiveness of our proposed model and methodology. Simulation findings reveal that, under varying electrolyzer capacities, ignoring the PEM electrolyzer’s dynamic characteristics can result in a deviation in system operating. Compared with the proposed method, it can reduce the equipment degradation speed by a maximum of 5.78 times.

## 1. Introduction

The escalating global energy demand, coupled with the dwindling reserves of fossil fuels, presents a formidable and pressing challenge to worldwide energy security [1]. In reaction to the looming climate change crisis, China has unveiled an ambitious strategy to peak its carbon dioxide emissions by 2030 and attain carbon neutrality by 2060 [2]. The power sector, at the forefront of carbon reduction initiatives, highlights the critical need for a low-carbon transition of power systems as a paramount national strategic objective [3]. Hydrogen, emerging as a highly adaptable energy carrier, exhibits exceptional versatility across a wide range of sectors, including power generation, chemical manufacturing, metallurgy, and transportation, thereby occupying a pivotal position in global efforts to curtail carbon emissions [4]. However, the exorbitant cost of hydrogen production through water electrolysis remains a substantial barrier to its widespread adoption. This obstacle can be overcome by generating hydrogen through water electrolysis driven by low-carbon electricity sourced from renewable energy (RE). This type of hydrogen can serve as a crucial raw material in various chemical processes, such as hydrogenation in refineries. Moreover, it can be combined with oxygen, nitrogen, and carbon to create compounds like methanol, ammonia, and methane. This strategy not only provides a feasible method for utilizing RE but also introduces an innovative route for facilitating a green and low-carbon transformation of power systems [5].

Electrolyzers serve as the cornerstone equipment for hydrogen generation, being classified into three primary types: PEM electrolyzers, alkaline electrolyzers, and solid oxide electrolyzers [6,7]. PEM electrolyzers, distinguished by their operational adaptability and swift response times, are particularly well-suited for integrating RE sources. Nevertheless, their advancement is impeded by elevated costs, intricate system designs, and stringent water quality requirements. Alkaline electrolyzers, representing a well-established water electrolysis technology, enjoy widespread usage in industrial settings [8]. Their primary limitations include reduced efficiency under partial load conditions and extended response times. Solid oxide electrolyzers, though promising, are not yet ready for large-scale industrial implementation [9]. Presently, PEM and alkaline electrolyzers are the predominant choices for industrial hydrogen production when powered by renewable sources. The selection between them depends on both technical and economic considerations. Technically, PEM electrolyzers offer flexible operation across a range of power levels, whereas alkaline electrolyzers are limited by minimum operational power thresholds. Economically, alkaline electrolyzers present significantly lower investment costs compared to PEM systems. Given the intricate dynamic characteristics of PEM electrolyzers, this study centers on optimizing their performance.

Traditionally, the cost of producing hydrogen through water electrolysis has remained elevated, largely attributable to the substantial electricity consumption inherent in the electrolysis process, which is commonly fueled by grid electricity derived from fossil fuels. Nonetheless, coupling electrolysis with low-carbon electricity sourced from RE such as wind, solar, or hydropower presents a viable approach to mitigating this cost challenge. Initially, renewable energy sources, notably solar and wind, frequently encounter intervals of low electricity demand or excess generation, wherein the marginal cost of electricity can approach zero. By harnessing this surplus energy for electrolysis, the operational expenses associated with hydrogen production can be notably diminished. Secondly, renewable energy initiatives, like solar or wind farms, typically incur lower long-term operational costs in comparison to fossil-fuel-dependent power generation, thereby further curtailing the cost of hydrogen production over an extended period. This strategy not only drives down the price of hydrogen but also fosters the establishment of a sustainable, low-carbon energy framework, simultaneously tackling both economic and environmental hurdles in the realm of hydrogen production.

In the optimal operation of IES, the efficiency and energy consumption ratios of PEM electrolyzers are frequently treated as fixed values. However, in actual practice, PEM efficiency is closely tied to its electrochemical properties and operational power levels [10,11], with hydrogen production efficiency potentially dropping by up to 20% under certain conditions [12]. This problem becomes even more pronounced when PEM electrolyzers are powered by intermittent and fluctuating ES sources. If the dynamic characteristics of PEM electrolyzers are overlooked and their efficiency and energy consumption ratios are still assumed to be constant, simulation results will be significantly skewed or overly optimistic, potentially leading decision-makers astray and resulting in flawed choices. Therefore, optimizing IES requires incorporating the dynamic characteristics of PEM electrolyzers and formulating more accurate technical–economic models. Existing research on PEM electrolyzer operation strategies highlights the importance of considering efficiency variations, operational load profiles, and transient start–stop behaviors in system optimization. Theoretically, PEM electrolyzers demonstrate higher efficiency at lower current densities, which translates to lower energy consumption per unit mass of hydrogen produced [13]. Moreover, PEM systems have an optimal operating range, typically spanning from 25% to 100% of their nominal capacity, with operation below this range posing a risk of overload [14]. These constraints may necessitate frequent start–stop cycles, underscoring the need for developing management protocols for PEM start–stop control. In summary, the current research gaps are as follows:

(1) The majority of existing research either concentrates on PEM models at the component or simulation level or assumes constant hydrogen production efficiency and energy consumption ratios in system-level operation models. These simplifications overlook the dynamic electrochemical responses of PEM electrolyzers under variable operating conditions, resulting in inaccurate estimations of hydrogen production and renewable energy utilization.
(2) The effects of frequent start–stop cycles, partial-load operations, and constrained operating ranges of PEM electrolyzers are frequently disregarded or merely represented through soft constraints. This approach tends to exaggerate operational flexibility and may lead to overly optimistic assessments of system-level economic and low-carbon performance.
(3) In numerous studies on electricity–hydrogen IES, the hydrogen network is either excluded or oversimplified to a “point-to-point” transfer model, without explicitly accounting for steady-state gas flow, node continuity, and pipeline resistance. This undermines the physical coherence between the electrical and hydrogen subsystems and can inaccurately depict the spatio-temporal interactions between electricity and hydrogen.

Existing studies treat PEM electrolyzers as static units. They ignore electrochemical polarization losses. They overlook start-up delays. This simplification distorts real operation. It overestimates hydrogen yield. It underestimates operating costs. We embed electrochemical dynamics into day-ahead scheduling. We capture current-dependent efficiency. We model discrete start–stop states. This approach maintains physical consistency. It bridges component-level electrochemistry and system-level economics.

Drawing on these insights, this paper delves into the dynamic behaviors of PEM electrolyzers within electricity–hydrogen integrated energy systems (IES). It formulates a model that captures the electrochemical influences on hydrogen production efficiency and integrates dynamic efficiency fluctuations. Building on this foundation, an optimized operational framework for integrated electricity–hydrogen systems is established. The key innovative contributions of this paper are outlined below:

(1) We develop a physics-aware PEM electrolyzer model that explicitly accounts for electrochemical polarization features, variable hydrogen production efficiency, and start–stop dynamics. Binary variables capture start–stop impacts. This model establishes a direct relationship between operating current, power consumption, and hydrogen production rate, embedding the dynamic attributes of PEM electrolyzers into the day-ahead operation of an electricity–hydrogen IES.
(2) Leveraging the dynamic PEM model, we construct an optimal operation model for an electricity–hydrogen IES aimed at minimizing daily operational costs, encompassing electricity procurement expenses, penalties for renewable energy curtailment, and carbon emission costs. We propose a novel Loss of Life Cost (LLC) model that quantifies the economic impact of operational cycles on PEM electrolyzer degradation. This model captures the degradation of proton exchange membranes and catalyst layers caused by frequent start–stop transitions and load variations, enabling total cost of ownership optimization.
(3) To improve computational efficiency, we introduce a model transformation method that linearizes the nonlinear relationships in the PEM electrolyzer model and the hydrogen pipeline gas flow equations through piecewise linear approximation. Along with a suitable simplification of the power flow equations, the entire problem is reformulated as a mixed-integer linear programing (MILP) model, which can be effectively solved using commercial optimization software.

## 2. Dynamic Characteristics of PEM Electrolysis Cells

PEM electrolyzers are frequently configured in parallel arrangements, wherein multiple electrolyzers are linked side by side to facilitate flexible and scalable hydrogen generation. In these systems, each electrolyzer functions as an independent entity, enhancing adaptability to fluctuations in renewable energy inputs—such as wind or solar power—and bolstering resilience against system malfunctions. The modular nature of parallel setups also allows for adjustable hydrogen production to align with diverse operational requirements. This configuration proves especially beneficial in IES that rely on intermittent renewable energy sources, as it ensures continuous electrolyzer operation even when one unit necessitates maintenance or repair. The modular strategy offers multiple advantages, including straightforward scalability, balanced load distribution among multiple units, and the capacity to effectively manage power variations inherent to variable renewable energy supplies. In this paper, the dynamic characteristics refers to the quasi-steady-state operational dynamics of PEM electrolyzers within the day-ahead scheduling framework, rather than fast electrochemical transients. Consequently, the model employs discrete-time-difference equations to capture inter-temporal operational constraints, ensuring accurate representation of electrolyzer flexibility while maintaining computational tractability for system-level optimization.

### 2.1. PEM-Based Water Electrolysis System for Hydrogen Production

**Figure 1:**
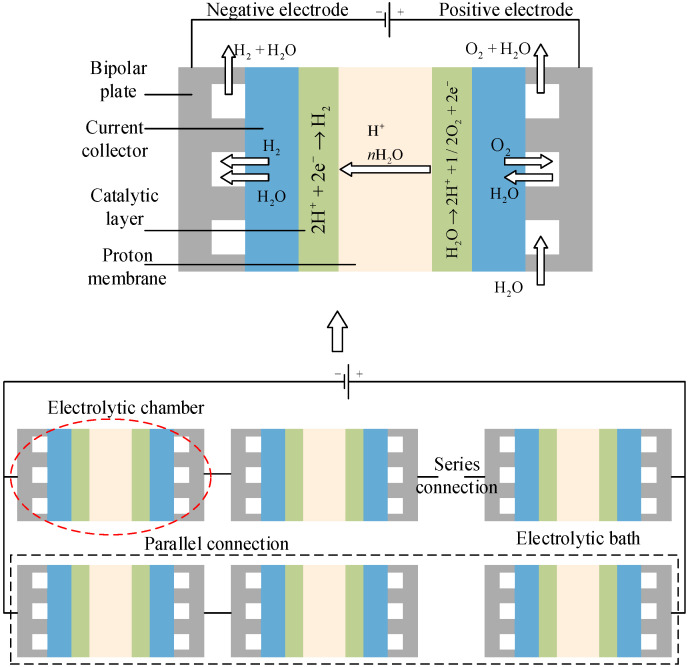
Design and fabrication of PEM-based water electrolysis system for hydrogen production.

Figure 1 depicts a parallel arrangement of PEM electrolyzers, in which each individual unit is linked to a shared power supply and works in tandem to produce hydrogen. This setup enables the effective allocation of electrical power and hydrogen output among multiple electrolyzers, enhancing both system reliability and scalability. The diagram also identifies crucial components, such as the electrolyzer stacks, current regulators, and the corresponding electrical and hydrogen interconnections.

As illustrated in Figure 1, a parallel configuration of multiple PEM electrolyzers facilitates hydrogen production by harnessing surplus RE. Each PEM electrolyzer comprises a series of current-regulated electrolysis cells operating under compression. By dynamically adjusting the current and implementing discrete start–stop cycles across the electrolyzers, the system can swiftly adapt to fluctuations in surplus ES, enabling flexible hydrogen output control [15,16,17,18]. Currently, water electrolysis technologies for hydrogen production mainly include PEM electrolyzers, alkaline electrolyzers, and anion exchange membrane (AEM) electrolyzers. The distinguishing features of each technology are summarized in Table 1.

The comparative analysis in Table 1 highlights the critical role of selecting electrolyzer technology by weighing both technical performance and economic viability. For example, while PEM electrolyzers entail higher upfront costs, they provide unmatched operational flexibility and significantly lower carbon emissions—especially when deployed in renewable energy-driven systems. Their capacity to quickly adapt to renewable energy variability enhances resource utilization and reduces environmental impact. These attributes not only improve the operational efficiency of IES but also underscore the unique value and innovation of the approach proposed in this study compared to conventional methods. The key contributions of this paper, in contrast to existing representative research, are summarized in Table 2 below.

The hydrogen generation rate of a PEM electrolyzer demonstrates a dynamic relationship with its operational conditions, especially during startup and shutdown phases. Under stable operating conditions, hydrogen production can be accurately controlled by adjusting the applied current density within the water electrolysis process. For individual cells within the electrolyzer stack, the polarization behavior—characterizing the relationship between open-circuit voltage $V_{c.t}^{\mathrm{PEM}}$ and current density $V_{c.t}^{\mathrm{PEM}}$—is mathematically defined as [19]:

(1) $$\begin{array}{c} V_{c.t}^{\mathrm{PEM}}=V_{0}+\frac{RT}{2F}\mathrm{In}\left(\frac{p_{H_{2}}\sqrt{p_{O_{2}}}}{p_{H_{2}O}}\right)+R_{c}^{\mathrm{PEM}}i_{c.t}^{\mathrm{PEM}}\\ +\frac{RT}{\alpha_{\mathrm{an}}F}\mathrm{arsinh}\left(\frac{i_{c.t}^{\mathrm{PEM}}}{2i_{\mathrm{an}}}\right)+\frac{RT}{\alpha_{\mathrm{cat}}F}\mathrm{arsinh}\left(\frac{i_{c.t}^{\mathrm{PEM}}}{2i_{\mathrm{cat}}}\right) \end{array}$$

where *F* represents the Faraday constant, *T* and *R* represent the temperature constant and the universal gas constant, respectively, *V*_0_ denotes the reversible cell voltage, $\alpha_{\mathrm{an}}$, $\alpha_{\mathrm{cat}}$, $I_{\mathrm{an}}$, and $i_{\mathrm{cat}}$ are the charge transfer coefficients and exchange current densities characterizing the electrode kinetics at the anode and cathode, $p_{H_{2}}$,$p_{O_{2}}$, and $p_{H_{2}O}$ represent the partial pressures of oxygen, hydrogen, and water vapor in the electrolysis gas stream, respectively, and $R_{c}^{\mathrm{PEM}}$ signifies the equivalent resistance.

### 2.2. Loss of Life Cost Modeling for PEM Electrolyzers

Frequent start–stop cycles and rapid load variations induce mechanical stress and chemical degradation in PEM electrolyzers, particularly affecting the proton exchange membrane and catalyst layers. To quantify this economic impact, we introduce the Loss of Life Cost (LLC) model.

Let $N_{c,t}^{start}$ and $N_{c,t}^{stop}$ represent the number of start and stop cycles for electrolyzer c in time period *t*, calculated via binary state variables $u_{c,t}$:

(2) $$N_{c,t}^{start}=\max(0,u_{c,t}-u_{c,t-1})$$

(3) $$N_{c,t}^{stop}=\max(0,u_{c,t-1}-u_{c,t})$$

The accumulated degradation $D_{c}$ is modeled as:

(4) $$D_{c}=\sum_{t=1}^{T}\left(\delta_{start}\cdot N_{c,t}^{start}+\delta_{stop}\cdot N_{c,t}^{stop}+\delta_{load}\cdot \frac{\left|P_{c,t}^{PEM}-P_{c,t-1}^{PEM}\right|}{P_{c}^{rated}}\right)$$

where $\delta_{start}$ and $\delta_{stop}$ represent the degradation per start/stop cycle and $\delta_{load}$ represents the degradation coefficient for load variations. The Loss of Life Cost is then calculated as:

(5) $$C_{LLC}=C_{cap}^{PEM}\cdot \sum_{c=1}^{N_{PEM}}\frac{D_{c}}{100}$$

where $C_{cap}^{PEM}$ is the capital cost of the PEM electrolyzer. This formulation transforms physical degradation into economic costs, enabling direct comparison with operational expenses in the objective function.

Under normal conditions, the parameters of each cell are identical. Therefore, if the open-circuit voltage $V_{c.t}^{\mathrm{PEM}}$ of each cell is equal, the current density $I_{c.t}^{\mathrm{PEM}}$ of each electrolysis system remains consistent. Based on the structure of the electrolyzer, the consumed ES $P_{c.t}^{\mathrm{PEM}}$ and the produced hydrogen $F_{c.t}^{\mathrm{PEM}}$ can be expressed as functions of the operating current $V_{c.t}^{\mathrm{PEM}}$ and the number of units in operation $I_{c.t}^{\mathrm{PEM}}$, as shown below [20]:

(6) $$P_{c.t}^{\mathrm{PEM}}=k_{1}N_{c}^{\mathrm{PEM}}V_{c.t}^{\mathrm{PEM}}A_{c}^{\mathrm{PEM}}I_{c.t}^{\mathrm{PEM}}$$

(7) $$F_{c.t}^{\mathrm{PEM}}=\frac{k_{2}\eta P_{c.t}^{\mathrm{PEM}}}{2Fk_{1}V_{c.t}^{\mathrm{PEM}}}$$

where *k*_1_ and *k*_2_ are conversion coefficients, *η* is the Faraday efficiency, $N_{c}^{\mathrm{PEM}}$ is the number of electrolyzers, and $A_{c}^{\mathrm{PEM}}$ is the membrane contact area.

Based on Equations (1)–(3), by further taking into account the start–stop status $U_{c.t}^{\mathrm{PEM}}$ of the electrolyzers, the nonlinear relationship between $P_{c.t}^{\mathrm{PEM}}$ and $F_{c.t}^{\mathrm{PEM}}$ can be expressed as:

(8) $$F_{c.t}^{\mathrm{PEM}}=\left\{\begin{array}{ll} 0, & U_{c.t}^{\mathrm{PEM}}=0\\ \frac{k_{2}\eta P_{c.t}^{\mathrm{PEM}}}{2Fk_{1}V_{c.t}^{\mathrm{PEM}}}, & U_{c.t}^{\mathrm{PEM}}=1 \end{array}\right.$$

To maintain safe operating conditions of the electrolyzer system throughout its lifecycle, $P_{c.t}^{\mathrm{PEM}}$ needs to be constrained within its upper bound $P_{c.\max}^{\mathrm{PEM}}$ and lower bound $P_{c.\min}^{\mathrm{PEM}}$ as follows:

(9) $$U_{c.t}^{\mathrm{PEM}}P_{c.\mathrm{lo}}^{\mathrm{PEM}}\leq P_{c.t}^{\mathrm{PEM}}\leq U_{c.t}^{\mathrm{PEM}}P_{c.\mathrm{up}}^{\mathrm{PEM}}\;U_{c,t}^{PEM}-U_{c,t-1}^{PEM}\leq \nu_{c,t}^{up},\;U_{c,t-1}^{PEM}-U_{c,t}^{PEM}\leq \nu_{c,t}^{down},\;\forall c,\forall t\geq 1$$

The above equation defines the feasible operating power range at each single interval and explicitly characterizes the temporal state transition dynamics by coupling the on/off status between consecutive time steps *t* and *t* − 1. This ensures that the start-up and shut-down actions are modeled as time-coupled discrete events, capturing the inter-temporal operational dynamics of PEM electrolyzers within the day-ahead scheduling horizon.

From the aforementioned formulas, it can be concluded that the hydrogen production efficiency of the PEM electrolyzer is variable and can be derived as:

(10) $$\eta_{c.t}^{\mathrm{PEM}}=\frac{m_{H_{2}}F_{c.t}^{\mathrm{PEM}}}{k_{3}P_{c.t}^{\mathrm{PEM}}}=\frac{m_{H_{2}}\eta }{2Fk_{4}V_{c.t}^{\mathrm{PEM}}}$$

where *k*_3_ and *k*_4_ are conversion coefficients and $m_{H_{2}}$ represents the higher heating value (HHV) of hydrogen. From the above equation, it can be seen that, when $m_{H_{2}}$ is higher, the PEM electrolyzer operates at a higher level.

## 3. Optimization Operation Model for Electricity–Hydrogen IES

Building upon the previously developed PEM electrolyzer model, this section integrates operational constraints specific to the combined electricity–hydrogen energy system. Aiming to minimize daily operational expenditures—which include carbon emission costs, energy consumption charges, and penalties for unused wind/solar power—an optimized operation model for the electricity–hydrogen IES is formulated. This model explicitly accounts for the dynamic behaviors of the PEM electrolyzer throughout its operation.

### 3.1. Basic Structure of the Electricity–Hydrogen IES

Hydrogen is widely recognized as the most promising secondary energy carrier due to its advantageous storage properties, enabling it to effectively complement electricity and play a pivotal role in shaping a future low-carbon society. To achieve this vision, we propose an integrated electricity–hydrogen energy system that leverages both electrical and hydrogen-based energy vectors to seamlessly accommodate high-penetration renewable ES [21,22,23].

**Figure 2:**
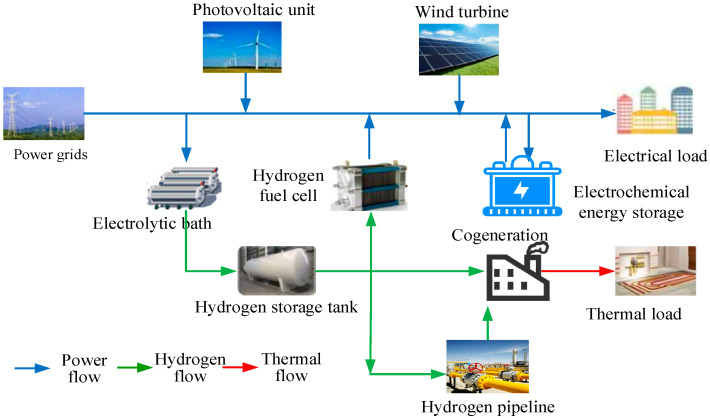
Construction of electricity–hydrogen IES.

Figure 2 presents the structural framework of the integrated electricity–hydrogen energy system proposed in this study, which strategically harnesses hydrogen’s carbon-neutral attributes to meet diverse energy needs—including electricity, heating, and hydrogen fuel. This is achieved by integrating photovoltaic panels and wind turbines for sustainable power generation, alongside a hybrid ES system combining lithium-ion batteries for short-term power stabilization and compressed hydrogen storage via PEM electrolysis for long-term energy balancing. The electrolyzer-storage integration is optimized to maximize hydrogen production during periods of renewable energy surplus, supplying both fuel cell vehicles and stationary power units while recovering waste heat through a CHP system. This system is further supplemented by a gas boiler to ensure reliable domestic hot water provision under fluctuating load conditions. The primary workflow of the methodology outlined in this paper is depicted below.

The optimization of the PEM electrolyzer in this study focuses primarily on enhancing its electrochemical performance and operational efficiency, which are mathematically represented as functions of current density, voltage, and energy input/output dynamics. Since the optimization framework is specifically tailored to the electrolyzer’s operation under predefined power conditions, energy transformations between different forms—such as those occurring in the broader electricity–hydrogen integrated system—do not directly affect the optimization process. By design, the electrolyzer’s operation is decoupled from these broader energy conversions, treating input electrical energy and hydrogen output as independent variables optimized according to efficiency metrics and operational constraints. This approach avoids the complexities of upstream or downstream energy conversion processes, ensuring that the electrolyzer’s optimization remains unaffected by inter-energy form transformations within the system.

### 3.2. Objective Function

Taking into account the dynamic operational behavior of the PEM electrolyzer, the objective function for developing an optimized operation model of the IES is formulated as follows:

(11) $$\min\;C_{total}=\sum_{t=1}^{T}\left(\begin{array}{l} c_{curt}\cdot P_{cur,t}+c_{grid}\cdot P_{grid,t}\\ -c_{RDG}\cdot P_{RDG,t}+c_{trad}\cdot e_{car}\cdot P_{grid,t} \end{array}\right)+C_{LLC}$$

where *c_curt_*, *c_grid_*, *c_RDG_*, and *c_trad_* represent the penalty price for curtailed wind/solar power, electricity purchase price, ES feed-in tariff, and carbon trading price, respectively. *e_car_* is the carbon emission factor. $C_{LLC}$ is the Loss of Life Cost, representing the economic depreciation of PEM electrolyzers due to operational cycling. The carbon emission cost represents indirect emissions from grid electricity purchases.

### 3.3. Constraints

At the constraint formulation stage, the operational constraints of the IES must align with the foundational principles governing both electrical power and hydrogen systems, while accounting for the unique operational characteristics of each interconnected component. This requires establishing tailored model constraints for the power grid, hydrogen distribution network, and their coupled units. By systematically integrating these sub-model constraints, a comprehensive set of operational constraints for the entire electricity–hydrogen IES can be derived. Notably, the constraints specific to the PEM electrolyzer have been detailed in prior sections. Below, we provide an in-depth analysis of key constraints, including power flow equations, operational limits for hydrogen storage tanks and fuel cells, hydrogen pipeline flow dynamics, network-wide flow continuity conditions, constraints on electrochemical energy storage systems, cogeneration unit operational boundaries, system-wide power balance requirements, and safety-related constraints for voltage and pressure management.

(1) Power Flow Equations

The power flow equations for the electrical grid describe the relationship between power injection and voltage levels and can be mathematically expressed as follows:

(12) $$P_{i}-U_{i}\sum_{j=1}^{N_{e}}U_{j}\left(G_{ij}\cos\theta_{ij}+B_{ij}\sin\theta_{ij}\right)=0$$

(13) $$Q_{i}-U_{i}\sum_{j=1}^{N_{e}}U_{j}\left(G_{ij}\sin\theta_{ij}-B_{ij}\cos\theta_{ij}\right)=0$$

where *P_i_* represents the injected active power; *Q_i_* represents the injected reactive power; *U_i_* and *U_j_* represent the voltage magnitudes; *θ_i_* and *θ_j_* are the voltage angles at nodes *i* and *j*, respectively; *G_ij_* and *B_ij_* represent the conductance and susceptance between nodes *i* and *j*, respectively; and *N_e_* is the total number of electrical nodes.

(2) Hydrogen Storage Tank Operational Constraints

(14) $$HyS_{t+1}=HyS_{t}+M_{\mathrm{ch}.t}-M_{\mathrm{dis}.t}$$

(15) $$HyS_{\min}\leq HyS_{t}\leq HyS_{\max}$$

(16) $$HyS_{t=1}=\alpha HyS_{\max}$$

(17) $$\Delta HyS_{\min}\leq HyS_{t}-HyS_{t-1}\leq \Delta HyS_{\max}$$

(18) $$M_{\mathrm{HyS}.\min}\leq M_{\mathrm{ch}.t}\leq Z_{\mathrm{HyS}}M_{\mathrm{HyS}.\max}$$

(19) $$M_{\mathrm{HyS}.\min}\leq M_{\mathrm{dis}.t}\leq (1-Z_{\mathrm{HyS}})M_{\mathrm{HyS}.\max}$$

In the equations, *HyS_t_* is the hydrogen mass stored in the tank and *m*_ch_._t_ and *m*_dic_._t_ are the hydrogen charging and discharging rates, respectively. HyS_min_ and HyS_max_ denote the lower and upper limits of the tank. *α* and *Z_HyS_* are the initial state coefficient and hydrogen charging/discharging state of the hydrogen storage tank. Δ*HyS*_max_ and Δ*HyS*_min_ are the ramp rate limits for hydrogen storage, while *m*_HyS_._min_ and *m*_HyS_._max_ denote the lower and upper limits for hydrogen charging and discharging of the tank. Equation (10) describes the storage balance of the hydrogen storage tank, and Equations (11)–(15) represent the storage capacity constraints and the upper and lower ramp rate constraints for the hydrogen storage tank.

(3) Hydrogen Fuel Cell Operational Constraints

(20) $$P_{t}^{HFC}=\eta_{e}^{HFC}m_{t}^{HFC}$$

(21) $$P_{\min}^{HFC}\leq P_{t}^{HFC}\leq P_{\max}^{HFC}$$

(22) $$\Delta P_{\min}^{HFC}\leq P_{t}^{HFC}-P_{t-1}^{HFC}\leq \Delta P_{\max}^{HFC}$$

where *m_t_^HFC^*, *η_t_^HFC^*, and *P_t_^HFC^* represent the hydrogen consumption, energy conversion efficiency coefficient, and electrical power output of the hydrogen fuel cell at time *t*, respectively. $P_{max}^{HFC}$, $P_{min}^{HFC}$ denote the maximum and minimum hydrogen consumption (or if referring to power output limits, they should be clearly labeled as such; here, we assume they refer to consumption limits for clarity), while Δ$P_{max}^{HFC}$ and Δ$P_{min}^{HFC}$ are the upper and lower ramp rate constraints of the fuel cell. Equation (16) describes the energy conversion process of the hydrogen fuel cell, and Equations (17) and (18) represent the capacity constraints and the upper and lower ramp rate constraints of the hydrogen fuel cell, respectively.

(4) Hydrogen Pipeline Gas Flow Equation

Considering the steady-state, isothermal flow of hydrogen through a pipeline, the relationship between the volumetric flow rate and the pressure differential along the pipeline can be expressed as:

(23) $$f_{r}=\psi \left(\Delta p_{r}^{2}\right)=K_{r}s_{ij}\sqrt{s_{ij}\left(p_{i}^{2}-p_{j}^{2}\right)}$$

where *f_r_* represents the hydrogen flow rate for pipeline *r*; *ψ* is a function symbol; $\Delta p_{r}^{2}=p_{i}^{2}-p_{j}^{2}$ denotes the pressure drop for pipeline *r*; *i* and *j* represent the node numbers at the two ends of pipeline *r*; *s_ij_* is a sign function used to describe the orientation of hydrogen movement within the pipeline; when *P_i_* > *P*_j_, *s_ij_* = 1; otherwise, *s_ij_* = −1; *K_r_* is the pipeline resistance coefficient for pipeline *r*, which can be expressed as:

(24) $$K_{r}=C_{n}\frac{D_{g}^{8/3}}{\sqrt{L_{g}T_{g}SZ_{a}}}$$

where *D_g_* represents the inner diameter of the pipeline; *L_g_* is the length of the pipeline; *T_g_* denotes the average temperature of the gas; *S* is the specific gravity of the gas; Z*_a_* is the average compressibility factor of the gas; and *C_n_* is a constant related to the reference temperature, reference pressure, and the universal constant for ideal gases.

(5) Equation for the Continuity of Hydrogen Flow in the Network

Setting $\Pi_{i}=p_{i}^{2}$, $\Delta \Pi_{i}=p_{r}^{2}$. The pressure drop matrix ΔΠ for hydrogen pipelines can be expressed as:

(25) $$A_{g}f=G$$

(26) $$-A_{g}^{T}\Pi =\Delta \Pi$$

where ***A****_g_* denotes the node-branch incidence matrix for the hydrogen network; ***f*** represents the vector containing the hydrogen flow rates in every pipeline; and ***G*** signifies the hydrogen flow rate at each individual node.

Combining the aforementioned equations, the pipeline flow equation for the hydrogen network can be expressed as:

(27) $$A_{g}\psi \left(-A_{g}^{T}\Pi \right)=G$$

(6) Electrical ES Operation Constraints

(28) $$SOC_{t+1}=SOC_{t}+(P_{\mathrm{ch}.t}-P_{\mathrm{dis}.t})$$

(29) $$SOC_{\min}\leq SOC_{s,t}\leq SOC_{\max}$$

(30) $$SOC_{t=1}=\beta SOC_{\max}$$

(31) $$P_{\mathrm{bat}.\min}\leq P_{\mathrm{ch}.t}\leq Z_{\mathrm{bat}}P_{\mathrm{bat}.\max}$$

(32) $$P_{\mathrm{bat}.\min}\leq P_{\mathrm{dis}.t}\leq (1-Z_{\mathrm{bat}})P_{\mathrm{bat}.\max}$$

where *SOC_t_*, *P_dis_*_,*t*_, and *P_ch_*_,*t*_ are the stored energy, discharging and charging power of the electrical ES system at time *t*, respectively. *SOC_min_* and *SOC_max_* are the lower and upper limits of the ES capacity. *P_bat_*_,*min*_ and *P_bat_*_,*max*_ are the lower and upper limits of the power for the electrical ES system. *β* and *Z_bat_* represent the initial state coefficient. Equation (24) describes the ES process of the electrical ES system, Equations (25) and (26) represent the SOC constraints, and Equations (27) and (28) represent the charging and discharging power constraints.

(7) Operational Constraints for combined heat and power (CHP) Units

The CHP unit integrates a micro-combustor with a bromine-based cooling system, where high-temperature steam generated by the combustor is processed through the bromine cooler to provide both heating and hot water. This configuration enables efficient, tiered utilization of thermal energy across different quality levels while reducing emissions of carbon dioxide and other pollutants. The mathematical formulation for this CHP unit is outlined below [18,19]:

(33) $$Q_{t}^{\mathrm{CHP}}=\eta_{\mathrm{rec}}\lambda_{\mathrm{he}}^{\mathrm{Co}}P_{t}^{\mathrm{CHP}}\left(1-\eta_{t}^{\mathrm{MT}}-\eta_{L}^{\mathrm{MT}}\right)/\eta_{t}^{\mathrm{MT}}$$

(34) $$\begin{array}{l} \eta_{t}^{\mathrm{MT}}=a_{\mathrm{MT}}{\left(P_{t}^{\mathrm{CHP}}\right)}^{3}+b_{\mathrm{MT}}{\left(P_{t}^{\mathrm{CHP}}\right)}^{2}+\\ c_{\mathrm{MT}}P_{t}^{\mathrm{CHP}}+d_{\mathrm{MT}} \end{array}$$

(35) $$G_{t}^{\mathrm{CHP}}=P_{t}^{\mathrm{CHP}}/\eta_{t}^{\mathrm{MT}}LH_{\mathrm{gas}}$$

In the equations, $P_{t}^{\mathrm{CHP}}$, $Q_{t}^{\mathrm{CHP}}$ and $G_{t}^{\mathrm{CHP}}$ represent the electrical power, thermal power, and gas consumption of the CHP system, respectively; $\eta_{t}^{\mathrm{MT}}$ is the power generation efficiency; *η*_rec_ and $\lambda_{\mathrm{he}}^{\mathrm{Co}}$ are the heating coefficient of the bromine cooler and the flue gas recovery efficiency, respectively; $\eta_{L}^{\mathrm{MT}}$ is the heat loss coefficient of the turbine; *a*_MT_, *b*_MT_, *c*_MT_ and *d*_MT_ are coefficients obtained through linear fitting of a polynomial to establish a linear model for the turbine efficiency.

(8) Power Balance Constraints for Integrated Electricity–Hydrogen Energy Systems

(36) $$\begin{array}{l} P_{RDG.t}+P_{\mathrm{dis}.t}+P_{t}^{\mathrm{CHP}}+P_{t}^{\mathrm{FC}}+P_{\mathrm{grid}.t}-\\ P_{t}^{\mathrm{Load}}-P_{\mathrm{cur}.t}-P_{t}^{\mathrm{PEM}}-P_{\mathrm{ch}.t}=\sum_{j\in \Omega_{\mathrm{bus}.i}}P_{ij.t} \end{array}$$

(37) $$Q_{\mathrm{grid}.t}+Q_{RDG.t}-Q_{t}^{\mathrm{Load}}=\sum_{j\in \Omega_{\mathrm{bus}.i}}Q_{ij.t}$$

(38) $$P_{\mathrm{cur}.t}\geq 0$$

(39) $$Q_{t}^{CHP}=H_{t}^{load}$$

where *P_RDG_._t_* and *P_cur_._t_* represent the active power generated by RE sources and the curtailed active power, respectively. *Q_RDG_._t_* and $Q_{t}^{\mathrm{Load}}$ denote the reactive power generated by RE sources and the reactive power demand of the load, respectively. *P*_grid_.*_t_* and *Q*_grid_.*_t_* represent the active and reactive power exchanged with the upstream power grid, respectively. $H_{t}^{Load}\;\mathrm{and}\;P_{t}^{\mathrm{Load}}$ represent the demands for thermal load and electrical load, respectively.

(9) Node Voltage and Gas Pressure Constraints

(40) $$U_{i.\min}\leq U_{i.t}\leq U_{i.\max}$$

(41) $$p_{i.\min}\leq p_{i.t}\leq p_{i.\max}$$

In the equations, *U_i_._min_* and *U_i_._max_* represent the lower and upper limits of the amplitude of the node voltage, respectively; *p_i_._max_* and *p_i_._min_* denote the upper and lower limits of the amplitude of the node gas pressure, respectively.

(10) Branch Transmission Capacity Constraints

(42) $$P_{ij.\min}\leq P_{ij.t}\leq P_{ij.\max}$$

(43) $$f_{r.\min}\leq f_{r.t}\leq f_{r.\max}$$

where *P_ij_._max_* and *P_ij_._min_* represent the transmission capacity limits of branch *ij*; *f_r_*._max_ and *f_r_.*_min_ denote the transmission capacity limits of pipeline *r*.

## 4. Model Transformation and Solution

The constraint Equations (1)–(6) governing the PEM electrolyzer, along with the power flow Equations (8) and (9) and hydrogen pipeline gas flow Equation (19) in the presented model, are all formulated as mixed-integer nonlinear constraints, which pose significant challenges for direct solution. To address this complexity, this study introduces a linearization transformation approach to reformulate the model into an MILP framework.

For the PEM electrolyzer constraint Equations (1)–(6), the functional relationship between $P_{c.t}^{\mathrm{PEM}}$ and $F_{c.t}^{\mathrm{PEM}}$ can be described as follows: when $U_{c.t}^{\mathrm{PEM}}$ = 1, $F_{c.t}^{\mathrm{PEM}}$ is a monotonically nonlinear function of $P_{c.t}^{\mathrm{PEM}}$, denoted as *f*_PEM_ ($P_{c.t}^{\mathrm{PEM}}$); otherwise, $F_{c.t}^{\mathrm{PEM}}$ is a linear function of $P_{c.t}^{\mathrm{PEM}}$, which can be represented by a straight line. Based on this, we divide the curve *f*_PEM_($P_{c.t}^{\mathrm{PEM}}$) into *N_p_* linear segments in the operative range of $P_{c.t}^{\mathrm{PEM}}$, transforming Equations (1)–(6) into:

(44) $$\begin{array}{c} F_{c.t}^{\mathrm{PEM}}=U_{c.t}^{\mathrm{PEM}}f_{\mathrm{PEM}}\left(P_{c.1}^{\mathrm{PEM}}\right)+\\ \sum_{k=1}^{N_{p}}\left[f_{\mathrm{PEM}}\left(P_{c.k+1}^{\mathrm{PEM}}\right)-f_{\mathrm{PEM}}\left(P_{c.k}^{\mathrm{PEM}}\right)\right]\theta_{c.k.t} \end{array}$$

(45) $$P_{c.t}^{\mathrm{PEM}}=U_{c.t}^{\mathrm{PEM}}P_{c.1}^{\mathrm{PEM}}+\sum_{k=1}^{N_{p}}\left(P_{c.k+1}^{\mathrm{PEM}}-P_{c.k}^{\mathrm{PEM}}\right)\theta_{c.k.t}$$

(46) $$\theta_{c.k+1.t}\leq \varphi_{c.k.t}\leq \theta_{c.k.t}\;k=1,2,\ldots ,N_{p}-1$$

(47) $$0\leq \theta_{c.k.t}\leq U_{c.t}^{\mathrm{PEM}}\;k=1,2,\ldots ,N_{p}$$

where $P_{c.k}^{\mathrm{PEM}}$ represents the value of the *k*-th segment point in the operative range of $P_{c.t}^{\mathrm{PEM}}$. *θ_c_._k_._t_* and *φ_c_._k_._t_* are the continuous auxiliary variables and binary auxiliary variables, respectively.

Likewise, the hydrogen pipeline gas flow equations can be reformulated using a piecewise linearization technique, resulting in the following expression [16]:

(48) $$f_{r.t}^{\mathrm{abs}}=K_{r}^{2}\left(\Pi_{i.t}-\Pi_{j.t}\right)$$

(49) $$f_{r.t}^{\mathrm{abs}}\approx \left|\zeta_{mn.t}^{1}\right|\zeta_{mn.t}^{1}+\sum_{k=1}^{N_{mn}}\left(\left|\zeta_{mn.t}^{k+1}\right|\zeta_{mn.t}^{k+1}-\left|\zeta_{mn.t}^{k}\right|\zeta_{mn.t}^{k}\right)\varphi_{k.t}$$

(50) $$f_{r.t}=\zeta_{mn.t}^{1}+\sum_{k=1}^{N_{mn}}\left(\zeta_{mn.t}^{k+1}-\zeta_{mn.t}^{k}\right)\varphi_{k.t}$$

(51) $$\varphi_{k+1.t}\leq \delta_{k.t}\leq \varphi_{k.t},\forall k\in N_{mn}-1$$

(52) $$0\leq \varphi_{k.t}\leq 1,\forall k\in N_{mn}$$

where $\zeta_{mn.t}^{1}$ represents the value of the *k*-th segment point in the operative range of *f_r_._t_*. *φ_k_._t_* and *δ_k_._t_* are continuous auxiliary variables and binary auxiliary variables, respectively.

For the power flow equations, considering two characteristics of the distribution network, (1) the node voltage magnitudes are close to 1 p.u. and (2) the phase angle differences between the two ends of the lines are very small, the power flow equations can be transformed into [16,17]:

(53) $$P_{i}=\sum_{j=1}^{N_{e}}\left[G_{ij}\left(U_{i}+U_{j}-1\right)+B_{ij}\theta_{ij}\right]$$

(54) $$Q_{i}=\sum_{j=1}^{N_{e}}\left[-B_{ij}\left(U_{i}+U_{j}-1\right)+G_{ij}\theta_{ij}\right]=0$$

## 5. Case Study Analysis

### 5.1. Simulation Case Setup

This paper performs a simulation-based analysis of an integrated electricity–hydrogen energy system, which integrates a 24-node power grid with a 20-node hydrogen distribution network [24,25]. To evaluate the efficacy of the proposed optimization approach—accounting for the dynamic behaviors of PEM electrolyzers—three distinct operational strategies are designed and tested. The model developed in this paper is solved using Gurobi on a computing platform equipped with an Intel Core i7-12700H processor and 32 GB of RAM. The scenarios are outlined as follows:

Scenario 1: The dynamic characteristics of the PEM electrolyzer are neglected, and hydrogen production efficiency is treated as a constant parameter. The operational strategy for the integrated electricity–hydrogen system is formulated accordingly [25].

Scenario 2: The operational power range constraints of the PEM electrolyzer are omitted, assuming the electrolyzer can start and stop instantaneously with full flexibility [26]. However, to demonstrate the economic impact of degradation, the LLC is calculated post-optimization based on the resulting aggressive cycling profile.

Scenario 3: The operational strategy for the integrated electricity–hydrogen energy system is developed using the proposed model, which explicitly incorporates the dynamic characteristics of the PEM electrolyzer and integrates the LLC into the objective function, enabling degradation-aware optimization.

**Figure 3:**
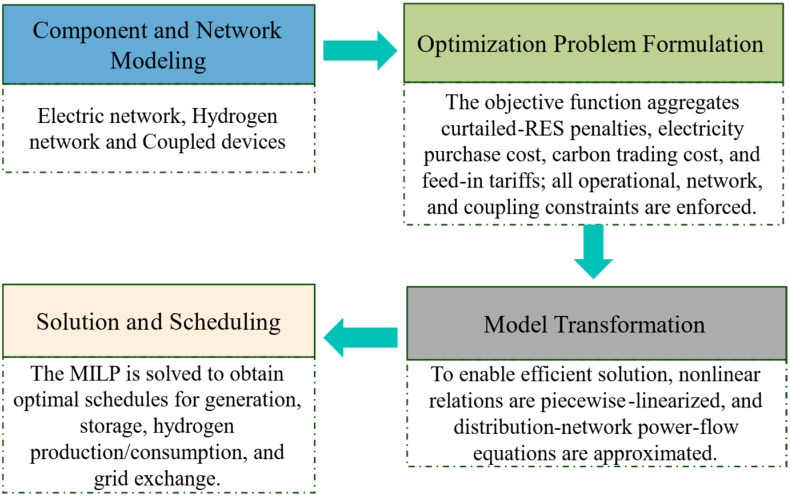
Flowchart of the method.

Table 3 outlines the equipment specifications for the IES. The variability profiles of RE sources and load demands are sourced from [26], with additional technical and economic parameters provided in [27,28]. Operational strategies for the system are formulated under three distinct scenarios. Table 2 evaluates and compares the economic performance across these scenarios, while Table 3 examines differences in hydrogen production and power consumption resulting from various PEM electrolyzer modeling approaches. Figure 3 illustrates the system carbon emissions and $P_{c.t}^{\mathrm{PEM}}$ consumption across scenarios, and Table 4 provides a sensitivity analysis on the impact of PEM electrolyzer capacity on system operational costs.

### 5.2. Comparative Analysis of Simulation Results

(1) Economic Performance Analysis

Table 4 summarizes the operational economic results across Scenarios 1–3. The findings indicate that, in Scenario 1, the costs linked to wind/solar curtailment, electricity purchases, and carbon emissions are substantially higher than in Scenarios 2 and 3, leading to the least favorable economic performance. This highlights that ignoring the dynamic characteristics of PEM electrolyzers results in significant renewable energy curtailment, compromising optimal utilization of renewable resources and reducing overall system economic efficiency. In comparison to Scenario 3, Scenario 2 achieves a 5.63% reduction in total operational costs, with lower expenses related to curtailment, carbon emissions, and electricity procurement. However, this improvement is based on an overestimation of PEM electrolyzer flexibility, which inaccurately reflects actual ES consumption and carbon emissions, skewing assessments of system performance. Conversely, Scenario 3, which incorporates the dynamic characteristics of PEM electrolyzers, enables more precise modeling of hydrogen production. It avoids errors in estimating curtailment and carbon emissions, leading to better integration of energy storage. This demonstrates that accounting for dynamic electrolyzer behavior improves the accuracy of system operational evaluations, supporting more dependable and cost-efficient decision-making.

The findings indicate that Scenario 3 achieves the low degradation cost of ownership when LLC is incorporated. While Scenario 2 shows lower operational costs, its frequent cycling incurs prohibitive degradation costs, making it economically inferior to Scenario 3. Notably, Scenario 1—which is representative of conventional approaches—overestimates system efficiency. This validates that ignoring dynamic characteristics and degradation leads to significant cost estimation errors.

(2) Hydrogen Production Analysis

Table 5 provides a comparative analysis of hydrogen production performance across different PEM electrolyzer modeling approaches. As illustrated in Table 3, while Scenario 1 consumes more electrical energy, its hydrogen output is notably lower than that in Scenarios 2 and 3. This discrepancy arises because Scenario 1 neglects the dynamic characteristics of the PEM electrolyzer, leading to inaccurate modeling of the relationship between hydrogen production power consumption and output. Consequently, the electrolyzer’s operational state is misrepresented, resulting in unreliable simulations of the system’s hydrogen production capacity. In contrast, Scenario 2 assumes the electrolyzer has unlimited operational flexibility, allowing it to adaptively generate hydrogen under varying conditions and achieve maximum hydrogen energy production. However, in real-world applications, electrolyzer performance is limited by factors such as start–stop cycles and electrochemical reaction kinetics, which restrict truly flexible operation across all scenarios. Scenario 3, which incorporates the dynamic characteristics of the PEM electrolyzer, enables precise modeling of hydrogen production processes. This ensures an accurate representation of the relationship between hydrogen output and power consumption, accounting for actual operational constraints and improving simulation reliability. Hydrogen functions as an internal energy carrier, primarily used to store surplus renewable energy. This role helps reduce curtailment penalties and facilitates the efficient integration of renewable energy into the system, ultimately contributing to overall cost optimization.

As illustrated in Table 5, Scenario 1 consumes more electrical energy but produces less hydrogen due to the constant efficiency assumption failing to capture partial-load inefficiencies. Scenario 2 assumes unrealistic flexibility, overestimating hydrogen production by 4.4% while subjecting the equipment to severe degradation. Scenario 3, incorporating electrochemical dynamics and LLC optimization, achieves balanced operation with minimal degradation, ensuring sustainable long-term operation.

**Figure 4:**
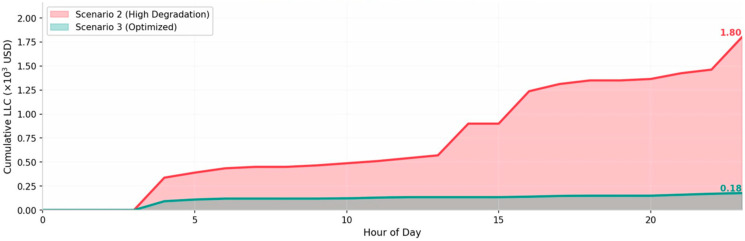
Cumulative Loss of Life Cost.

Figure 4 shows the degradation costs in different scenarios, with Scenario 2 having significantly higher degradation costs due to frequent switching compared to the proposed method.

### 5.3. Sensitivity Analysis

Table 6 provides a comparative analysis of total system operational costs across different electrolyzer capacities. The findings indicate that, for all scenarios, total costs decline steadily as electrolyzer capacity increases. This trend occurs because greater electrolyzer capacity boosts hydrogen production, which in turn enhances ES utilization, reduces carbon emissions, and improves overall economic efficiency. Furthermore, the cost differences between Scenarios 1–2 and Scenario 3 become more pronounced with rising electrolyzer capacity. This divergence arises because Scenarios 1–2 do not accurately account for the dynamic behavior of PEM electrolyzers. As capacity grows, hydrogen production increases, amplifying inaccuracies in modeling the hydrogen production process. As a result, renewable energy consumption, carbon emissions, and wind/solar curtailment are significantly misestimated, leading to a larger discrepancy in operational cost accuracy.

To contextualize these insights, Table 7 compares the proposed strategy with representative optimized approaches commonly employed in the existing literature.

In contrast to models that assume constant efficiency or ignore operational constraints, our PEM electrolyzer-aware approach incorporates variable efficiency, start–stop limitations, load-range restrictions, and a physically accurate hydrogen network representation within an MILP framework. This methodology effectively avoids overly optimistic or pessimistic evaluations of system operating costs. Ultimately, the proposed strategy provides enhanced accuracy and dependability for operational decision-making processes.

### 5.4. Robustness Analysis

To evaluate the influence of RE source intermittency on the model, a sensitivity analysis was performed with increased variability. The resulting system operational costs are presented in Table 8.

As illustrated in Table 8, greater RE intermittency increases operational costs across all scenarios. However, the proposed model exhibits superior robustness. High-capacity electrolyzers effectively buffer renewable variability, maintaining stable operational costs.

### 5.5. Comparison of Various Models

To validate the proposed electrochemical model, we compare simulation results with experimental data from Stansberry et al. [10] and Hernandez-Gomez et al. [11] for a 60kW PEM electrolyzer under varying load conditions.

**Figure 5:**
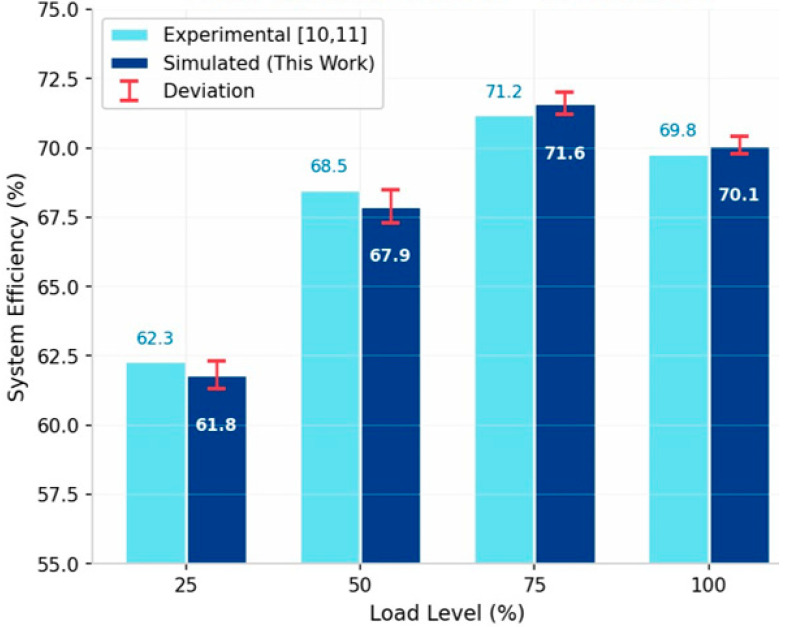
Model validation against experimental data compared with Stansberry et al. [10] and Hernan-dez-Gomez et al. [11].

As shown in the Figure 5, the maximum absolute deviation is 0.9%, confirming the accuracy of the electrochemical modeling. Table 9 contextualizes the proposed strategy against representative approaches in the recent literature using similar system scales.

In contrast to models assuming constant efficiency or ignoring operational constraints, our PEM electrolyzer-aware approach incorporates variable efficiency, explicit degradation costs, and physically accurate hydrogen network representation. This methodology achieves 6.5% lower costs than [13] and 4.7% lower than [16]. The inclusion of LLC prevents the optimistic bias inherent in unconstrained models, providing more reliable decision-making for long-term asset management.

## 6. Conclusions

This research integrates the dynamic behaviors and degradation economics of PEM electrolyzers into an operational framework for integrated electricity–hydrogen energy systems. By utilizing electrolyzers to efficiently absorb surplus RE while accounting for the Loss of Life Cost (LLC) induced by operational cycling, the proposed method reduces both system carbon emissions and instances of wind/solar curtailment, while ensuring asset longevity. Simulation findings reveal that, under varying electrolyzer capacities, ignoring the PEM electrolyzer’s dynamic characteristics can result in a deviation in system operating. Compared with the proposed method, it can reduce the equipment degradation speed by a maximum of 5.78 times.

In the future, we will explore multi-timescale optimization integrating day-ahead scheduling with real-time degradation monitoring and investigate advanced membrane materials to reduce cost.

## Figures and Tables

**Table 1:** Comparative technology table for electrolyzers.

Technology	Efficiency	Capital Cost	Carbon Emissions	Flexibility
PEM Electrolyzers	High (70–80%)	High	Low	High
Alkaline Electrolyzers	Moderate (55–65%)	Moderate	Moderate	Low
AEM Electrolyzers	Moderate (60–70%)	Low	Moderate	Moderate

**Table 2:** The main contribution of this paper compared to existing references.

Aspect	Existing Representative Work	the Main Contributions of this Paper
PEM model	Experiments or mechanism modeling are mostly conducted at the component or simulation level or employ constant efficiency at the system level.	Exploring the dynamic characteristics of PEM electrolysis cells, incorporating variable efficiency and start–stop impacts into system-level intraday operations.
Impact of Start–Stop and Load Range	This metric is often overlooked or approximated with soft constraints.	This paper employs explicit binary variables to characterize the impact of start–stop operation and power intervals, thereby avoiding the optimistic bias.
H2 network	Most references do not explicitly model or only calculate “point-to-point” traffic.	This paper investigates the characteristics of isothermal steady-state flow in relation to node continuity and pipe resistance, maintaining physical consistency between the electricity–hydrogen dual networks.

**Table 3:** Rated capacities of electricity–hydrogen IES equipment.

Node	Equipment	Total Capacity
1,4,8,10	Hydrogen Storage Tank	3000 kg
2,6,15,20	PEM Electrolyzer	200 MW
16,23	Hydrogen Fuel Cell	200 MW
11,17	CHP Plant	280 MW
2,5,10	Wind Farm	500 MW
18,23	PV Power Plant	640 MW
7,9,16	Electrical ES System	500 MWh

**Table 4:** Comparisons of operation results under different cases.

Scenario	C_curt_	C_grid_	C_carbon_	C_LLC_
1 (Constant Efficiency)	10.98	12.79	3.45	0.00
2 (Unconstrained)	4.23	9.45	2.56	6.85
3 (Proposed model)	5.67	10.67	2.75	1.21

**Table 5:** Comparisons of hydrogen energy condition under different cases.

Scenario	Power Consumption (p.u.)	Hydrogen Production (p.u.)	Daily LLC (%)
1	780	650	0.00
2	650	715	2.43
3	679	685	0.42

**Table 6:** Comparison of system operation cost under different capacities.

Electrolyzer Capacity	Scenario 1	Scenario 2	Scenario 3
200 MW	36.67	35.67	30.92
260 MW	25.47	20.15	21.02
300 MW	21.03	14.34	19.79

**Table 7:** Comparative summary against existing optimized strategies.

Strategy	Key PEM Modeling & Constraints	Best Features/Limitations
Constant-efficiency PEM baseline (Scenario 1)	PEM efficiency treated as constant; dynamics ignored	Feature: simple and fast to model. Limitation: overestimates H_2_ from variable conditions; inflates curtailment and cost; misjudges emissions.
Idealized unconstrained PEM (Scenario 2)	Ignores practical start–stop/power-range limits	Feature: lower nominal costs. Limitation: physically unrealistic; systematically underestimates purchases/emissions; not directly implementable; Accelerate equipment degradation speed.
Proposed strategy (Scenario 3)	Variable efficiency; explicit start–stop and power-range constraints via binary variables; piecewise linearization of PEM characteristics	Features: physically consistent dispatch; avoids optimistic bias; tractable day-ahead scheduling; Reasonably control the degradation speed of equipment. Limitations: slightly higher cost than idealized unconstrained variant because realism is enforced.

**Table 8:** Comparison of system operation cost under high RES intermittency.

Electrolyzer Capacity	Scenario 1	Scenario 2	Scenario 3
200 MW	37.95	41.56	31.38
260 MW	26.23	24.18	21.44
300 MW	21.56	18.36	20.09

**Table 9:** Comparative summary against existing optimized strategies.

Model	PEM Model	LLC Considered	Total Cost
Zheng et al. [13]	Constant Efficiency	No	32.4
Li et al. [16]	Dynamic Efficiency	No	31.8
Proposed model	Consider Dynamic Efficiency and Equipment Degradation	Yes	30.3

## Data Availability

The authors confirm that the data supporting the findings of this study are available within the article.
